# Perchlorate Removal in Microbial Electrochemical Systems With Iron/Carbon Electrodes

**DOI:** 10.3389/fchem.2019.00019

**Published:** 2019-01-24

**Authors:** Qiao Yang, Fengxiang Zhang, Jingjing Zhan, Chao Gao, Minhui Liu

**Affiliations:** School of Food and Environment, Dalian University of Technology, Panjin, China

**Keywords:** microbial electrochemical systems, perchlorate removal, Fe/C, cathode, coulombic efficiency

## Abstract

Perchlorate removal was tested in the cathode chamber of microbial electrochemical systems (MESs). Dual-chambers MESs were constructed and operated in batch mode with four kinds of cathode materials including Fe/C particles (Fe/C), zero valent iron particles (ZVI), blank carbon felt (CF), and active carbon (AC). Without external energy supply or perchlorate-reducing microbial pre-enrichment, perchlorate (${\text{ClO}}_{4}^{-}$) removal could be achieved in the cathode chambers of MESs at different efficiencies. The highest ${\text{ClO}}_{4}^{-}$ removal rates in these reactors were 18.96 (Fe/C, 100 Ω, 2 days), 15.84 (ZVI, 100 Ω, 2 days), 14.37 (CF, 100 Ω, 3 days), and 19.78 mg/L/day (AC, 100 Ω, 2 days). ${\text{ClO}}_{4}^{-}$ degradation products were mainly Cl^−^ and ${\text{ClO}}_{3}^{-}$, and the total chlorine in the products was lower than the theoretical input. The non-conservation of the total chlorine may be caused by the adsorption and co-precipitation related to the electrode materials. Coulombs and coulombic efficiency calculation showed that electron provided by MESs was partially responsible for ${\text{ClO}}_{4}^{-}$ reduction, for the Fe/C cathode reactors, about a quarter of electron was provided by MESs.

## Introduction

Perchlorate is a kind of persistent chemicals included in the U.S. EPA contaminant candidate list. The aerospace fuels, explosives, and firecrackers could cause perchlorate contamination existing in the soil, groundwater, and drinking water. The perchlorate concentration can reach 3,700 mg/L in groundwater, 120 mg/L in surface water, and 811 μg/L in drinking water (Guan et al., 2015). It has been found in milk, urine, and blood (Cheng et al., 2004; Valentínblasini et al., 2005). Perchlorate adversely affects human health by interfering with the production of thyroid hormone (Wang et al., 2014). It has certain toxic effects on the development of the nervous system and the reproductive system. The environment and health problems caused by perchlorate have raised extensive concern.

Traditional perchlorate degradation processes are mainly activate carbon adsorption (Mahmudov and Huang, 2010) and ion exchange technology (Yoon et al., 2009), However, these techniques are limited to a low adsorption rate and the generation of chlorinated byproducts (Oh et al., 2005; Xiong et al., 2007). Previous study investigated the enrichment of perchlorate-reducing microbial communities in the cathode of the MESs, with the enrichment duration < 1 month. The perchlorate removal rate was 9.46 mg/L/day (Mieseler et al., 2013). Perchlorate was reduced without exogenous electron shuttles or fixed electrode potential, and Butler et al. (2010) had achieved a maximum perchlorate removal rate of 24 mg/L/day and cathode conversion efficiency of 84%. Perchlorate reduction in biological reactions was believed to occur via the sequential reduction ${\text{ClO}}_{4}^{-}$ - ${\text{ClO}}_{3}^{-}$ - ${\text{ClO}}_{2}^{-}$ - Cl^−^ (Wolterink et al., 2005). Iron powder is a kind of common reductant, which is harmless to health and widely applied in groundwater remediation. When temperature was increased from 25 to 40, 60, and 75°C progressively, rate constants were 0.013, 0.10, 0.64, and 1.52 mg perchlorate per gram nanoparticles per hour. The activation energy of perchlorate and iron reaction was calculated to be 79.02 ± 7.75 kJ/mole, and perchlorate reduction was limited by the slow kinetics (Cao et al., 2005). Lang et al. (2005) proved that perchlorate was initially adsorbed on the iron surface, followed by reduction to chloride. The electrostatic interaction, ion exchange, and surface complexation were conducive to the adsorption of perchlorate, among them the electrostatic attraction and ion exchange played a dominant role, accounting for 76% of perchlorate removal rate (Xu et al., 2013). In order to enhance the removal efficiency of perchlorate, investigators provided energy into the reaction system, and perchlorate could be completely degraded within 60 min at 195°C using Fe^2+^ (Gu et al., 2003). Increasing the reaction temperature by microwave technology could improve removal efficiency of perchlorate (Láng and Horányi, 2003; Horányi, 2004; Oh et al., 2005).

Previous research suggested that microorganism which could degrade perchlorate existed widely in environment. Although biological reduction of perchlorate showed great potential for large-scale perchlorate wastewater treatment, the enrichment of microorganisms requires relative long period of time. External energy could promote the reaction of ZVI and perchlorate under the anaerobic condition, and reduce the energy barrier of perchlorate reduction. However, providing extra energy to treat perchlorate may debase its practical significance (Im et al., 2011). As an environmental friendly technology, microbial electrochemical systems (MESs) can utilize various organic substrates to transform chemical energy directly into electricity. A series of contaminants have been catalyzed by microorganisms in the anode or reduced in the cathode (Ucar et al., 2017). Some studies have reported the use of MESs to treat perchlorate. Perchlorate was removed in the anode chamber with different electron donors (Lian et al., 2017). Following research has been focused on the perchlorate reduction using the biocathode of MESs (Shea et al., 2008; Butler et al., 2010; Mieseler et al., 2013; Li et al., 2015; Lian et al., 2016). As the carrier of biocathodes, the electrode materials used in these researches were graphite granules, polyaniline modified graphite, Pt-coated carbon cloth, or carbon fiber brushes.

In this study, consideration has been given to the effects of cathode materials on the perchlorate removal without perchlorate-reducing microbial pre-enrichment in MESs. The suitable cathodes of MESs may donate electrons for the perchlorate reduction reaction. Carbon was chosen in this study as their adsorption capacity and frequent use in MESs cathode. Iron material was also chosen as it has been tested in perchlorate degradation. We prepared iron and carbon materials as MESs cathodes, including Fe/C particles (Fe/C), ZVI particles (ZVI), blank carbon felt (CF), and active carbon powder (AC), and investigated perchlorate removal with the effect of different electrode materials in microbial electrochemical systems. Without adding external power or perchlorate-reducing microbial enrichment culture, the perchlorate degradation efficiencies have been compared and analyzed.

## Experimental Methods

### Electrode Materials

Carbon brushes of 3 cm diameters were used as the anodes after they were heated at 450°C for 30 min (Yang et al., 2017). Four kinds of cathode materials were exploited, including Fe/C, ZVI, CF, and AC. Fe/C particles (3~5 μm) were obtained by hydrothermal method and carbon thermal reduction with variation of the molar ratio of sucrose to FeCl_3_ (13:5) (Sun et al., 2009). ZVI (400 mesh), AC powder and carbon felt (≥99%, 1 mm thick) were commercially available. Blank carbon felt was pre-treated by boiling at 90°C H_2_O_2_ (10 % wt) for 3 h then soaking in deionized water for 1 h. Cathode was prepared according to the reference with some adaptation as the following procedures (Cheng et al., 2006). One kind of cathode particle (120 mg Fe/C, AC, or ZVI) was mixed with 400 μL sodium carboxymethyl cellulose, 200 μL Nafion solution, and 100 μL isopropanol in a 5 mL plastic sample vial, ultrasonic vibration was operated for 10 min. Both sides of blank carbon felt (D = 3.8 cm) were coated with the paste-like mixture in vial as even and homogenous a consistency as possible using a small paintbrush. Allow the coating to dry in N_2_ for 24 h. The loading of cathode particle was 5.29 mg per square centimeter carbon felt area.

### Reactors Construction and Operation

Dual-chambers MESs were constructed with pre-cultured carbon brush anodes and four kinds of cathodes, which were identified as Fe/C, ZVI, CF, and AC. Each reactor connected with a 100 or 1,000 Ω external resistance. The anode substance was 1 g/L sodium acetate and minimal trace in 50 mM phosphate buffer solution (28 mL). Catholyte was 50 mg/L ${\text{ClO}}_{4}^{-}$ solution (KClO_4_, 28 mL). The two chambers were separated by a proton exchange membrane (D = 3.8 cm, 32S, Hangzhou Grion, China). All reactors were operated in batch at room temperature (25~28°C). The anode and cathode chambers of MES were kept sealed during the whole experiment and the systems were in the condition of anaerobic/anoxic. Both the catholyte and anolyte were replaced every cycle and duration was 2, 3, or 6 days at different experimental notes.

### Analysis Methods

${\text{ClO}}_{4}^{-}$ and ionic byproducts such as chlorate (${\text{ClO}}_{3}^{-}$), chlorite (${\text{ClO}}_{2}^{-}$) and hypochlorite (ClO^−^), and Chloride (Cl^−^) in the solution were analyzed using a Dionex DX-600 ion chromatography system with an AS-23 auto sampler and a 4 × 250 mm analytical column. Eluent was 50 mmol NaOH solution with 1.0 mL/min inflow rate. Applied current was set to 110 mA and injection volume was 25 μL. Samples were filtered using 0.22 μm Nylon membrane filter before entering the ion chromatography. COD was measured using standard method (APHA, 1995). Current was obtained by I = U/R, while voltage was recorded using an external data acquisition system. Coulombs of organic substrate was calculated by C = FbSv/M, where F is Faraday's constant, b is 4 mol of electrons/mol of COD, S (g/L) the substrate concentration, v (L) the liquid volume, and M is 32 the molecular weight of O_2_ (Liu and Logan, 2004). Q_COD_ was obtained by subtracting coulombs of effluent from coulombs of influent. Total coulombs transmitted by the external circuit (Q_ex_) was equal to integration of current with respect to time. Coulombic efficiency (CE) was calculated by comparing the actual coulombs transmitted by the external circuit (Q_ex_) to the available coulombs (Q_COD_) according to the reference (Yang et al., 2012). Total coulombs used for perchlorate degradation (Q_p_) was calculated based on the number of electron transfer for every kind of chlorine-containing degradation products detected in the effluent. Fresh Fe/C powder and graded used Fe/C cathodes surfaces were analyzed by X-ray photoelectron spectra (XPS; ESCALAB^TM^ 250i, ThermoFisher, USA) with Al Kα as the X-ray source (500 μm spot size, 0.05 eV energy step size for C, O, Cl, and Fe elements while 1.00 eV energy step size for whole spectra). Surface area and pore size distribution of four kinds of fresh cathode materials were analyzed by nitrogen adsorption desorption isotherm used full automatic physical adsorption instrument (Autosorb-iQ-C; Quantachrome Instruments; USA) with N_2_ as analysis gas.

## Results and Discussion

### Perchlorate Removal in Different MES Reactors

**Figure 1:**
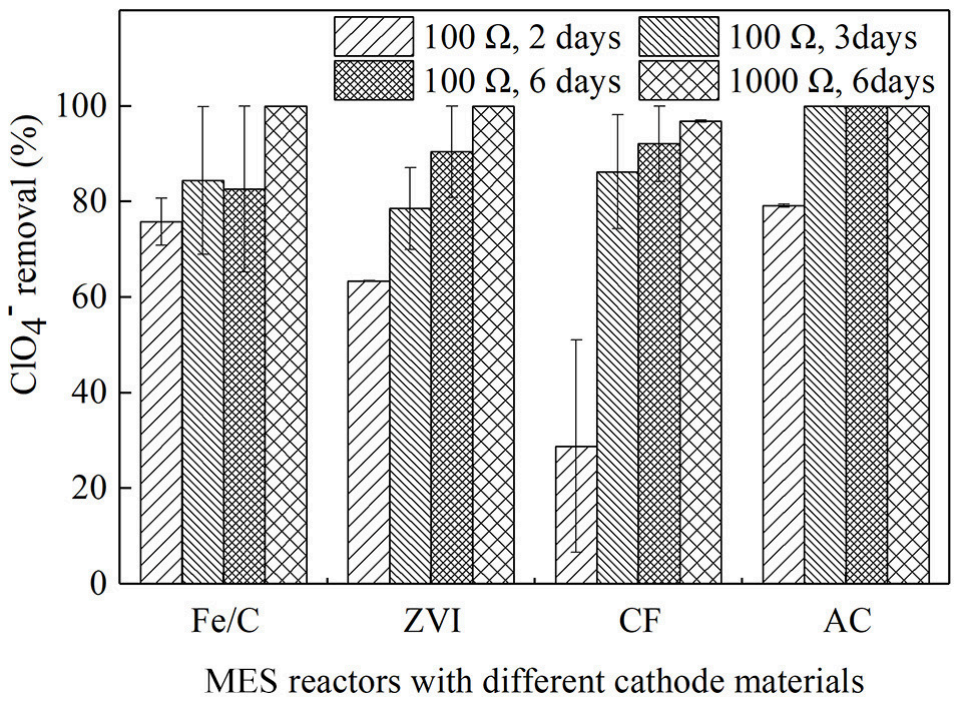
The removal efficiency of ${\text{ClO}}_{4}^{-}$ in MES reactors with different cathode materials at the different operation condition.

Operating duration of batch experiments and external resistance are factors that may affect the contaminant removal in MESs. The MESs used in this research were operated on batch-fed in cubic reactors, and output voltage would drop in 2 or 3 days as the anode substrate largely consumed based on preliminary studies, therefore 2 and 3 days were chosen as the operating duration. Six days was also chosen because the substrate in the anode champers would be exhausted by then, and the removal after 6 days could be considered as the highest removal when the anodic electron donors were consumed. When 100 Ω resistance was used in the external circuit, operation duration was set as 2, 3, or 6 days. External resistance, as an easy strategy to run MES, would significantly influence the current and anode performance (Zhang et al., 2017). 100 and 1,000 Ω were used in this research as the representatives of low resistance and high resistance. They were tested and compared in the systems when operating duration was set as 6 days. Reactors of Fe/C, ZVI, CF, and AC were operated for 2, 3, and 6 days with the external resistance of 100 Ω in proper order, then external resistances were turned into 1,000 Ω and batch duration last 6 days. The ${\text{ClO}}_{4}^{-}$ removal efficiency were showed in Figure 1. When batch operation duration was prolonged from 2 to 3 days and further to 6 days, the average removal efficiencies were kept increasing for ZVI (63.4 ± 0.0, 78.5 ± 8.6, and 90.4 ± 9.6%), CF (28.8 ± 22.2, 86.2 ± 12.0, and 92.1 ± 7.9%), and AC (79.1 ± 0.4, 100 and 100%) reactors, however in the Fe/C reactors the removal efficiencies after 3 and 6 days were similar (84.4 ± 15.5 and 82.6 ± 17.4%) and a little higher than that of 2 days (75.8 ± 4.9%). Then external resistances were changed from 100 to 1,000 Ω and operation duration was still kept 6 days, the removal efficiencies were further increased to 96.8 ± 0.2 (CF cathode reactors) and 100% (Fe/C, ZVI, and AC cathode reactors). Effective removal of ${\text{ClO}}_{4}^{-}$ was observed in the cathode chamber of MES with Fe/C, ZVI, CF, and AC as cathode materials, accompanied by numerical changes when cathode materials or operation conditions were changed.

When ${\text{ClO}}_{4}^{-}$ removal rates were calculated based on the same operating time, AC cathode reactors (19.78, 16.67, 8.33 mg/L/day) had higher removal rate than CF reactors (7.19, 14.37, 7.68 mg/L/day) when operation duration was 2, 3, and 6 days, respectively. In iron-contained cathode reactors, the ${\text{ClO}}_{4}^{-}$ removal rates for Fe/C (18.96, 14.06, 6.88 mg/L/day) and ZVI (15.84, 13.09, 7.53 mg/L/day) were decreasing when batch duration was prolonged. Cao et al. investigated perchlorate removal by nanoscale ZVI and found that 59.1% removal was obtained (initial concentration of 200 mg/L) at ZVI dosage of 20 g/L after 28 days, in which study the removal rate was 4.22 mg/L/d (Cao et al., 2005). The longest batch duration was 6 days in our research because the anode substrate would be consumed in several days in the batch operation. In the former bio-cathode MESs research, perchlorate reducing bacteria (PCRB) were inoculated on carbon brushes cathode, the running period was 14~17 days with 500 Ω resistance loaded, and ${\text{ClO}}_{4}^{-}$ removal was 37.3% after running 15 days. ${\text{ClO}}_{4}^{-}$ was not significant reduced in the control reactors of open circuit and non-bio-cathode, which indicated that the degradation of perchlorate was considered to be a biological reaction (Mieseler et al., 2013). In this research, there were no PCRB inoculated, and effective ${\text{ClO}}_{4}^{-}$ removal could still be obtained. To analyze the progress of ${\text{ClO}}_{4}^{-}$ removal, further analysis was approached.

### Perchlorate Removal Analysis Related to Electrode Materials

**Figure 2:**
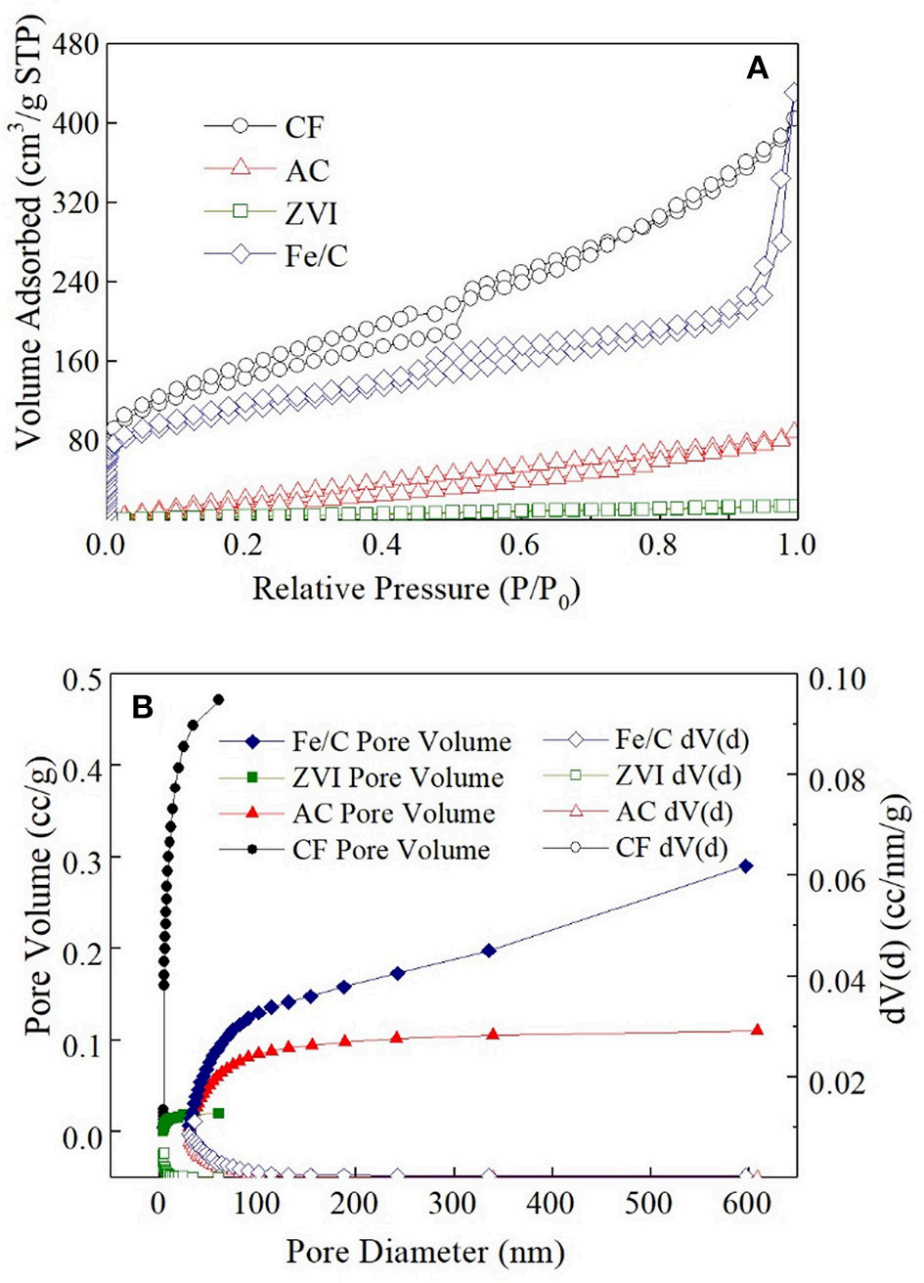
Nitrogen adsorption desorption isotherm **(A)** and pore size distribution **(B)** of four cathode materials.

AC powder is known as the adsorption material, and the other electrode materials may have adsorption effect on ${\text{ClO}}_{4}^{-}$. The surface area and pore size distribution of electrode materials used in this research were analyzed by Nitrogen adsorption desorption isotherm (Figure 2). The specific surface area of fresh CF after grinding was largest (491.07 m^2^/g), which was mainly composed of macro porous. The specific surface area of Fe/C particles was 381.42 m^2^/g, which was 2.8 times of that of AC powder (136.30 m^2^/g). AC particles had a large number of microporous and mesoporous structure and most of the Fe/C particles had mesoporous structure. The microporous AC had many adsorption sites and good removal effect on ${\text{ClO}}_{4}^{-}$ (Wu et al., 2012). The specific surface area of ZVI particles was 22.73 m^2^/g and nearly had no suitable adsorption pore structure. When 60 mg AC, ZVI and Fe/C particle were individually added into vials which filled with 20 mL ClO4- (50 mg/L) for 3days, 14.35 ± 0.35% ClO4- was removed from solution in AC vial, 7.05 ± 0.65% was removed in Fe/C vial and only 1.65 ± 1.45% was removed from ZVI vial. The adsorption results were only related to the pristine materials. When these materials were used as the cathodes, adsorption could be affected by other parameters. The removals were much lower than the results obtained in MES cathode chambers. The removal caused by the adsorption structure of the electrode material accounted for only a part of the total removal. The adsorption removal of perchlorate in Fe/C and ZVI systems were much lower than that in AC systems.

The removal of ${\text{ClO}}_{4}^{-}$ was not only an adsorption process, but also a redox reaction process. It has been reported that ${\text{ClO}}_{4}^{-}$ was adsorbed on the surface of iron material and then reduced to non-toxic Cl^−^ in the research about the electrochemical characteristics of perchlorate and iron group metals (Lang et al., 2005). In the Fe/C and ZVI cathode MES reactors, iron materials were used as the cathode materials, and ${\text{ClO}}_{4}^{-}$ could be adsorbed to the cathode surface then transformed into other products under the reduction of iron materials. In iron containing systems, ${\text{ClO}}_{4}^{-}$ could be reduced to Cl^−^ by Fe^0^ under anoxic conditions as ${\text{ClO}}_{4}^{-}$ + 4Fe^0^ + 8H^+^ → 4Fe^2+^ + Cl^−^ + 4H_2_O (Im et al., 2011). Fe^0^ was oxidized to Fe^2+^ and then further oxidized into Fe^3+^. Therefore, in Fe/C and ZVI reactors, iron contained cathode were consumable. The removal caused by co-precipitate happened in iron contained cathode systems (Fe/C and ZVI reactors), but not in AC and CF reactors. Also, this process might be pH dependent. The generation of hydroxides (e.g., ferrihydrite with high surface sites) when basic pH excursion occurs in the cathode chamber likely induces the adsorption and/or co-precipitation of ClO4- in the Fe/C and ZVI reactors, which, however, might not be observed in the pure adsorption experiments. The reaction between Fe(0) and water would result in the formation of hydroxides with much lower surface sites.

**Figure 3:**
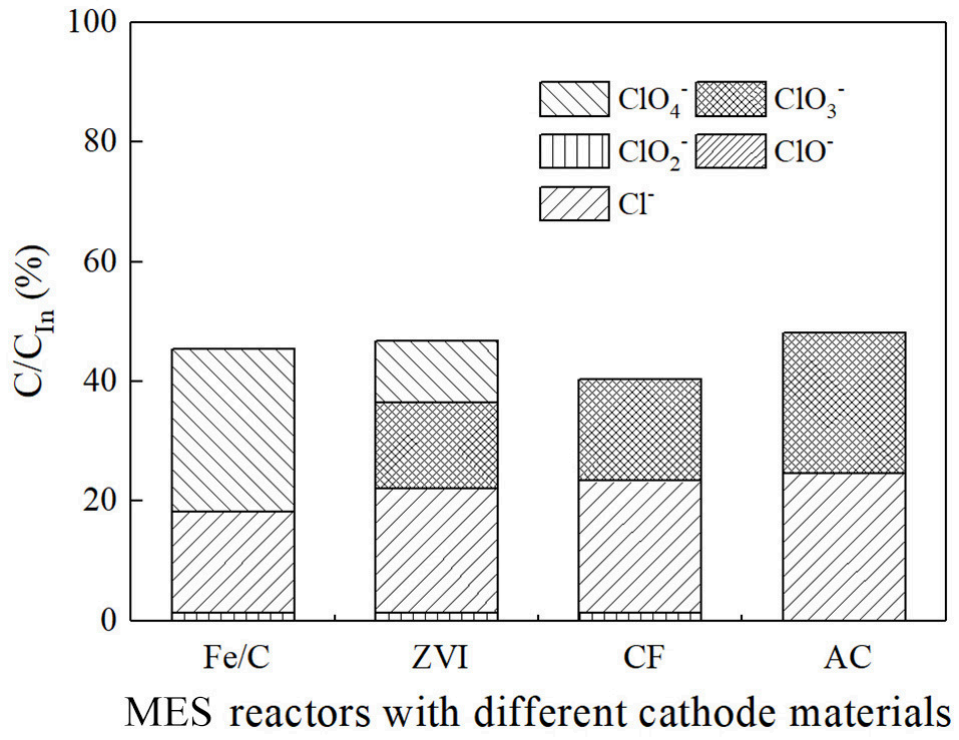
The ion percentage content analysis of effluent in different MES reactors.

**Figure 4:**
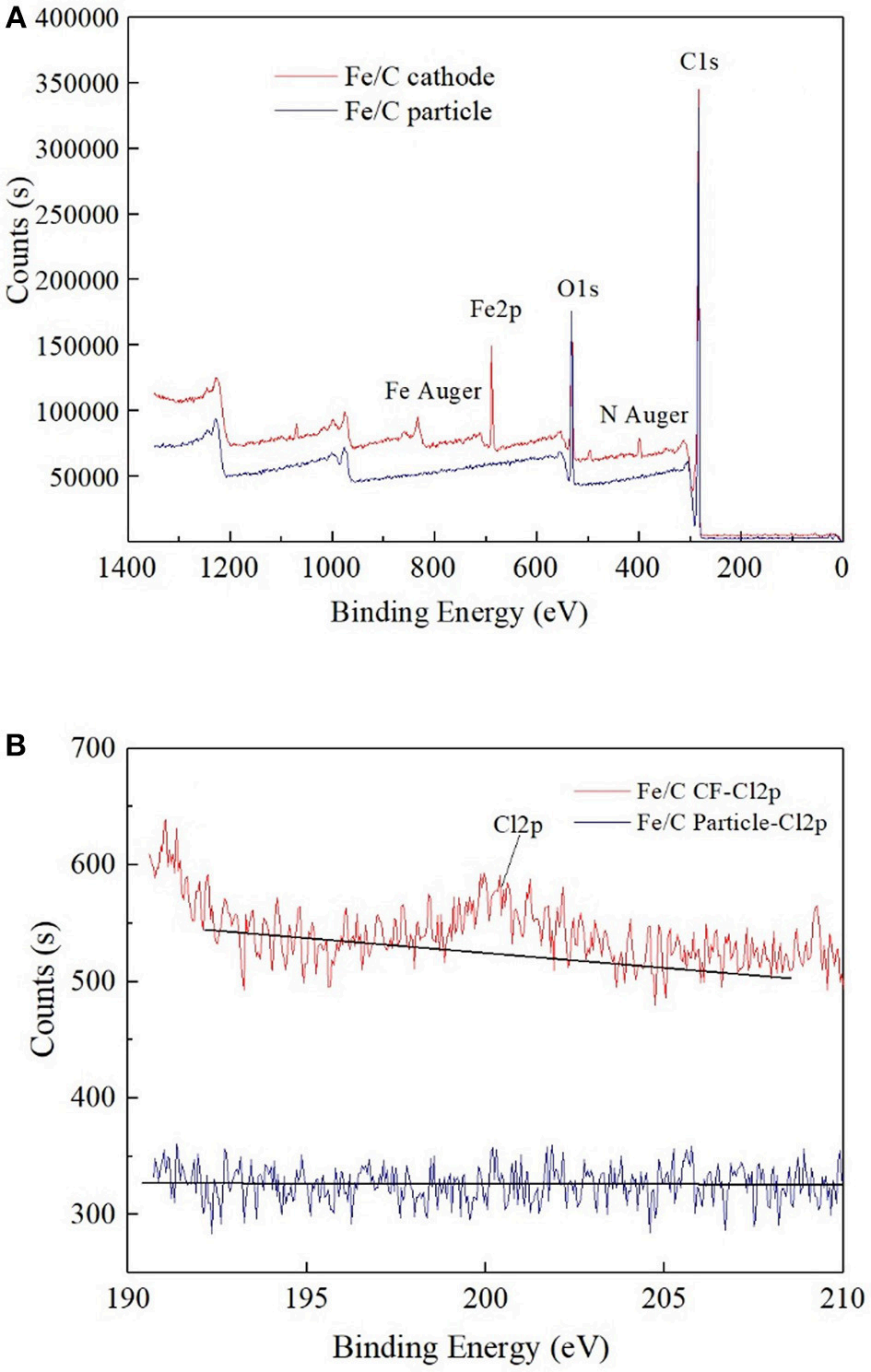
XPS analysis of used Fe/C cathode and fresh Fe/C particles: **(A)** Wide spectrum. **(B)** High-resolution Cl2p spectrum.

The ion percentage contents of effluents in every reactor were analyzed when 100 Ω external resistance was loaded for 3 days (Figure 3). Part of ${\text{ClO}}_{4}^{-}$ was reduced, and the products of ${\text{ClO}}_{4}^{-}$ reduction were mainly chloride ion and an amount of other intermediate products (${\text{ClO}}_{3}^{-}$, ${\text{ClO}}_{2}^{-}$, ClO^−^). The total chlorine content was less than half of theoretical value, which implied that a part of ${\text{ClO}}_{4}^{-}$ was not included in the measured effluent sample. This could be partly caused by the cathode material adsorption. In iron contained reactors, most of Fe(III) was in the form of hydroxide or oxide in alkaline cathode solution, which was normally positive charges and could co-precipitate negatively charged anions such as ${\text{ClO}}_{4}^{-}$, ${\text{ClO}}_{3}^{-}$, ${\text{ClO}}_{2}^{-}$, and Cl^−^ (Im et al., 2011). Therefore, part of chlorine related anions were wrapped in solid precipitation, which was attached to the cathode or filtrated by membrane filter before ion chromatograph injection. XPS analysis confirmed the existence of chloride on the surface of cathode. By comparing the fresh Fe/C particles (Fe/C particles) and used Fe/C carbon felt cathode (Fe/C cathode), the detailed XPS analysis indicated the existence of chloride after the Fe/C cathode was used in the ${\text{ClO}}_{4}^{-}$ removal system (Figure 4).

### Perchlorate Reduction by the Cathode of MES

**Figure 5:**
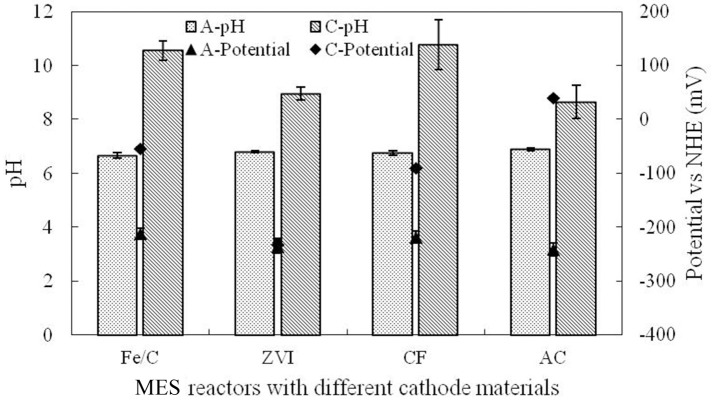
Electrode potential and pH in different reactors.

This research was operated in microbial electrochemical systems, and cathode potentials were higher than the anode potential by different degrees in different cathode reactors. Electrons of anode could be transferred to the cathode and ${\text{ClO}}_{4}^{-}$ could be reduced directly by the cathode. Electrode potential and pH were measured at the end of cycle, which are shown in Figure 5. Anolyte pH (A-pH) in each reactor was basically stable at neutral condition as PBS was used in anode chamber. Catholyte pH (C-pH) was increased at the end of cycle. Anode potential (A-potential) was basically stable at the normal biofilm potential, while the cathode potential (C-potential) showed obvious difference, which was associated with the catholyte environment and the cathode materials. Fe/C cathode potential was −54.1 mV, but the ZVI cathode potential was −232.4 mV, which was only a little bit higher than the anode. Cathode potential was the reflection of redox ability, which was related to the base material and catholyte. The perchlorate removal could be affected by the cathode potential.

Coulombs and coulombic efficiency (CE = Q_ex_/Q_COD_) of every reactor were calculated as Table 1. Fe/C and AC reactor had the highest coulombic efficiencies (over 32%). Coulombs that ${\text{ClO}}_{4}^{-}$ reduction required was calculated based on the respective quantities of production ${\text{ClO}}_{3}^{-}$, ${\text{ClO}}_{2}^{-}$, and Cl^−^. The number of Q_ex_/Q_p_ was from 5.92 to 24.35% (Table 1). The specific objective of this study was to compare the perchlorate removal and coulombs proportion provided by MES circuit (Q_ex_/Q_p_) when different cathode materials were used in MESs. The terminal electron donor was organic substrate in anode chamber of MES, so the higher of the number of Q_ex_/Q_p_, the more sustainability of the system. In an extremely ideal situation, when Q_ex_/Q_p_ is close to 100%, the cathode may be considered as virtually inexhaustible electron donor. In the present study, external circuit of Fe/C reactor had the biggest contribution to ClO4- reduction among all these reactors (24.35%), which number was more than 3 times higher than the smallest one (ZVI, 5.92%). The choice of cathode materials may affect the number of Q_ex_/Q_p_ significantly. Since the research was a preliminary research of perchlorate removal by Fe/C cathode in MESs, and external circuit coulombs functioned only a quarter of total reduction in current research, there is much room for improvement. Fe/C cathode may be more suitable for this system among the four kinds of materials and deserve more research. In previous literature, iron–carbon material have been proved to have synergistic adsorption and reduction abilities, which could be obtained by economic carbothermal reduction using only common sugar and ferrous sulfate as starting materials (Ai et al., 2016). The iron-carbon composites produced at low cost may have significant potential for the contaminant treatment or remediation. The iron contained in Fe/C electrode could be oxidized and influence the electrode repetitive use. After the Fe/C cathode MESs were operated for 6 continuous cycles at 100 Ω external resistance with the batch duration of 3 days, ${\text{ClO}}_{4}^{-}$ removal was decreased to 58%. It indicated that the Fe/C electrode was consumable electrode, which should be replaced regularly, and electrode corrosion caused by the salt deposition may also have a role. Electrode stability and repeatability should be considered in future research.

**Table 1:** Coulombs and coulombic efficiency calculation.

** 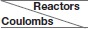 **	**Fe/C**	**ZVI**	**CF**	**AC**
Q_COD_ ^a^/ C	78.00	49.70	59.80	64.20
Q_ex_ ^b^/ C	25.40	8.70	13.40	21.10
CE/ %	32.56	17.50	22.40	32.87
Q_p_ ^c^/ C	104.30	147.00	159.60	181.60
Q_ex_/Q_p_ / %	24.35	5.92	8.40	11.61

a Q_COD:_ Coulombs of organic calculated by COD changes in anode.

b Q_ex:_ Total coulombs transmission of external circuit.

c *Q_p:_ Total coulombs used for perchlorate reduction*.

## Conclusion

Without additional energy input or PCRB inoculation, four kinds of chemical iron/carbon cathode in the dual-chambers MESs were used to degrade ${\text{ClO}}_{4}^{-}$. In iron containing reactors, the highest ${\text{ClO}}_{4}^{-}$ removal rates of Fe/C, Fe^0^ systems were 18.96, 15.84 mg/L/day. In non-iron containing systems, the highest ${\text{ClO}}_{4}^{-}$ removal rate of AC, CF systems were 19.78 and 14.37 mg/L/day under the condition of loading 100 Ω external resistance. Adsorption, co-precipitation and chemical reaction proceeded in these systems simultaneously. ${\text{ClO}}_{4}^{-}$ removal was the combination result of electrode adsorption, ZVI reduction, iron co-precipitation and cathode reduction. Calculation results showed that the MESs cathode reduction was only partially responsible for ${\text{ClO}}_{4}^{-}$ degradation, and that Fe/C had the highest coulombic efficiency (32.56%) and Q_ex_/Q_p_ (24.35%), which means coulombs proportion provided by MESs circuit was about a quarter of electrons needed in ${\text{ClO}}_{4}^{-}$ reduction. AC was wonderful adsorption material and had highest adsorption effect and perchlorate removal rate, but Fe/C cathode MESs had the highest Q_ex_/Q_p_. In this research, we did not just look for higher removal, but to find out suitable cathode materials. Fe/C cathode may be more suitable for MES system than other materials and deserve further research.

## Author Contributions

QY conceived the experiments. FZ carried out the experiment. QY and FZ wrote the manuscript. JZ participated in the Fe/C material preparation. CG and ML contributed to the discussion.

### Conflict of Interest Statement

The authors declare that the research was conducted in the absence of any commercial or financial relationships that could be construed as a potential conflict of interest.

## References

[B1] Ai L. He J. W. Wang Y. Y. Wei C. L. Zhan J. J. (2016). Aerosol-assisted *in situ* synthesis of iron-carbon composites for the synergistic adsorption and reduction of Cr(VI). RSC Adv. 6, 56108–56115. 10.1039/C6RA07953A

[B2] APHA (1995). Standard Methods for the Examination of Water and Wastewater. Washington, DC: American Public Health Association/American Water Works Association/Water Environment Federation.

[B3] Butler C. S. Clauwaert P. Green S. J. Verstraete W. Nerenberg R. (2010). Bioelectrochemical perchlorate reduction in a microbial fuel cell. Environ. Sci. Technol. 44, 4685–4691. 10.1021/es901758z 20476736

[B4] Cao J. S. Elliott D. Zhang W. X. (2005). Perchlorate reduction by nanoscale iron particles. J. Nanoparticle Res. 7, 499–506. 10.1007/s11051-005-4412-x

[B5] Cheng Q. Q. Perlmutter L. Smith P. N. McMurry S. T. Jackson W. A. Anderson T. A. (2004). A study on perchlorate exposure and absorption in beef cattle. J. Agric. Food Chem. 52, 3456–3461. 10.1021/jf049951c 15161215

[B6] Cheng S. Liu H. Logan B. E. (2006). Increased performance of single-chamber microbial fuel cells using an improved cathode structure. Electrochem. Commun. 8, 489–494. 10.1016/j.elecom.2006.01.010

[B7] Gu B. H. Dong W. J. Brown G. M. Cole D. R. (2003). Complete degradation of perchlorate in ferric chloride and hydrochloric acid under controlled temperature and pressure. Environ. Sci. Technol. 37, 2291–2295. 10.1021/es0262378 12785539

[B8] Guan X. Xie Y. Wang J. Wang J. Liu F. (2015). Electron donors and co-contaminants affect microbial community composition and activity in perchlorate degradation. Environ. Sci. Pollut. Res. 22, 6057–6067. 10.1007/s11356-014-3792-9 25382499

[B9] Horányi G. (2004). Investigation of the specific adsorption of HSO_4_^−^(${\text{SO}}_{4}{}^{2-}$) and Cl^−^ ions on Co and Fe by radiotracer technique in the course of corrosion of the metals in perchlorate media. Corr. Sci. 46, 1741–1749. 10.1016/S0010-938X(03)00315-9

[B10] Im J. K. Son H. S. Zoh K. D. (2011). Perchlorate removal in Fe^0^/H^2^O systems: impact of oxygen availability and UV radiation. J. Hazard Mater. 192, 457–464. 10.1016/j.jhazmat.2011.05.030 21705137

[B11] Lang G. Inzelt G. Vrabecz A. Horanyi G. (2005). Electrochemical aspects of some specific features connected with the behavior of iron group metals in aqueous perchloric acid/perchlorate media. J. Electroanal. Chem. 582, 249–257. 10.1016/j.jelechem.2005.01.006

[B12] Láng G. G. Horányi G. (2003). Some interesting aspects of the catalytic and electrocatalytic reduction of perchlorate ions. J. Electroanal. Chem. 552, 197–211. 10.1016/S0022-0728(02)01302-5

[B13] Li J. J. Gao M. M. Zhang G. Wang X. H. Wang S. G. Song C.. (2015). Perchlorate reduction in microbial electrolysis cell with polyaniline modified cathode. Bioresour. Technol. 177, 74–79. 10.1016/j.biortech.2014.11.065 25479396

[B14] Lian J. Tian X. L. Guo J. B. Guo Y. K. Song Y. Y. Yue L. (2016). Effects of resazurin on perchlorate reduction and bioelectricity generation in microbial fuel cells and its catalysing mechanism. Biochem. Eng. J. 114, 167–175. 10.1016/j.bej.2016.06.028

[B15] Lian J. Tian X. L. Li Z. F. Guo J. B. Guo Y. Z. Yue L. (2017). The effects of different electron donors and electron acceptors on perchlorate reduction and bioelectricity generation in a microbial fuel cell. Int. J. Hydr. Energy 42, 544–552. 10.1016/j.ijhydene.2016.11.027

[B16] Liu H. Logan B. E. (2004). Electricity generation using an air-cathode single chamber microbial fuel cell in the presence and absence of a proton exchange membrane. Environ. Sci. Technol. 38, 4040–4046. 10.1021/es0499344 15298217

[B17] Mahmudov R. Huang C. P. (2010). Perchlorate removal by activated carbon adsorption. Separ. Purif. Technol. 70, 329–337. 10.1016/j.seppur.2009.10.016

[B18] Mieseler M. Atiyeh M. N. Hernandez H. H. Ahmad F. (2013). Direct enrichment of perchlorate-reducing microbial community for efficient electroactive perchlorate reduction in biocathodes. J. Ind. Microbiol. Biotechnol. 40, 1321–1327. 10.1007/s10295-013-1318-y 23925794

[B19] Oh S. Y. Chiu P. C. Kim B. J. Cha D. K. (2005). Enhanced reduction of perchlorate by elemental iron at elevated temperatures. J. Hazard Mater. 129, 304–307. 10.1016/j.jhazmat.2005.09.006 16243433

[B20] Shea C. Clauwaert P. Verstraete W. Nerenberg R. (2008). Adapting a denitrifying biocathode for perchlorate reduction. Water Sci. Technol. 58, 1941–1946. 10.2166/wst.2008.551 19039173

[B21] Sun X. Zheng C. Zhang F. Yang Y. Wu G. Yu A. (2009). Size-controlled synthesis of magnetite (Fe_3_O_4_) Nanoparticles coated with glucose and gluconic acid from a single Fe(III) precursor by a sucrose bifunctional hydrothermal method. J. Phys. Chem. C 113, 16002–16008. 10.1021/jp9038682

[B22] Ucar D. Zhang Y. F. Angelidaki I. (2017). An overview of electron acceptors in microbial fuel cells. Front. Microbiol. 8:643. 10.3389/fmicb.2017.00643 28469607 PMC5395574

[B23] Valentínblasini L. Mauldin J. P. Maple D. Blount B. C. (2005). Analysis of perchlorate in human urine using ion chromatography and electrospray tandem mass spectrometry. Anal. Chem. 77, 2475–2481. 10.1021/ac048365f 15828783

[B24] Wang Z. Gao M. Zhang Y. She Z. Ren Y. Wang Z.. (2014). Perchlorate reduction by hydrogen autotrophic bacteria in a bioelectrochemical reactor. J. Environ. Manag. 142, 10–16. 10.1016/j.jenvman.2014.04.003 24794520

[B25] Wolterink A. Kim S. Muusse M. Kim I. S. Roholl P. J. van Ginkel C. G.. (2005). *Dechloromonas hortensis* sp. nov. and strain ASK-1, two novel (per)chlorate-reducing bacteria, and taxonomic description of strain GR-1. Int. J. Syst. Evol. Microbiol. 55, 2063–2068. 10.1099/ijs.0.63404-0 16166710

[B26] Wu C. D. Dou J. L. Xu X. H. Zhang B. (2012). Study on perchlorate wastewater treatment by coconut shell active carbon. Ind. Saf. Environ. Prot. 38, 15–17.

[B27] Xiong Z. Zhao D. Pan G. (2007). Rapid and complete destruction of perchlorate in water and ion-exchange brine using stabilized zero-valent iron nanoparticles. Water Res. 41, 3497–3505. 10.1016/j.watres.2007.05.049 17597179

[B28] Xu J. H. Gao N. Y. Deng Y. Xia S. Q. (2013). Nanoscale iron hydroxide-doped granular activated carbon (Fe-GAC) as a sorbent for perchlorate in water. Chem. Eng. J. 222, 520–526. 10.1016/j.cej.2012.07.141

[B29] Yang Q. Liang S. Liu J. Lv J. Feng Y. (2017). Analysis of anodes of microbial fuel cells when carbon brushes are preheated at different temperatures. Catalysts 7:312 10.3390/catal7110312

[B30] Yang Q. Wang X. Feng Y. Lee H. Liu J. Shi X. (2012). Electricity generation using eight amino acids by air–cathode microbial fuel cells. Fuel 102, 478–482. 10.1016/j.fuel.2012.04.020

[B31] Yoon I. H. Meng X. Wang C. Kim K. W. Bang S. Choe E.. (2009). Perchlorate adsorption and desorption on activated carbon and anion exchange resin. J. Hazard Mater. 164, 87–94. 10.1016/j.jhazmat.2008.07.123 18789577

[B32] Zhang L. Li J. Zhu X. Ye D. D. Fu Q. Liao Q. (2017). Startup performance and anodic biofilnn distribution in continuous-flow microbial fuel cells with serpentine flow fields: effects of external resistance. Ind. Eng. Chem. Res. 56, 3767–3774. 10.1021/acs.iecr.6b04619

